# BVLOS UAS Operations in Highly-Turbulent Volcanic Plumes

**DOI:** 10.3389/frobt.2020.549716

**Published:** 2020-10-27

**Authors:** Kieran Wood, Emma J. Liu, Tom Richardson, Robert Clarke, Jim Freer, Alessandro Aiuppa, Gaetano Giudice, Marcello Bitetto, Kila Mulina, Ima Itikarai

**Affiliations:** ^1^Department of Aerospace Engineering, University of Bristol, Bristol, United Kingdom; ^2^Department of Earth Sciences, University College London, London, United Kingdom; ^3^School of Geographical Sciences, University of Bristol, Bristol, United Kingdom; ^4^University of Saskatchewan Centre for Hydrology, Canmore, AB, Canada; ^5^Dipartimento di Scienze della Terra e del Mare, University of Palermo, Palermo, Italy; ^6^Istituto Nazionale di Geofisica e Vulcanologia, Osservatorio Etneo, Sezione di Catania, Catania, Italy; ^7^Rabaul Volcanological Observatory, Rabaul, Papua New Guinea

**Keywords:** unmanned aircraft system (UAS), UAV, aerial robotic, volcano, plume, BVLOS, Manam, gas sensing

## Abstract

Long-range, high-altitude Unoccupied Aerial System (UAS) operations now enable *in-situ* measurements of volcanic gas chemistry at globally-significant active volcanoes. However, the extreme environments encountered within volcanic plumes present significant challenges for both air frame development and in-flight control. As part of a multi-disciplinary field deployment in May 2019, we flew fixed wing UAS Beyond Visual Line of Sight (BVLOS) over Manam volcano, Papua New Guinea, to measure real-time gas concentrations within the volcanic plume. By integrating aerial gas measurements with ground- and satellite-based sensors, our aim was to collect data that would constrain the emission rate of environmentally-important volcanic gases, such as carbon dioxide, whilst providing critical insight into the state of the subsurface volcanic system. Here, we present a detailed analysis of three BVLOS flights into the plume of Manam volcano and discuss the challenges involved in operating in highly turbulent volcanic plumes. Specifically, we report a detailed description of the system, including ground and air components, and flight plans. We present logged flight data for two successful flights to evaluate the aircraft performance under the atmospheric conditions experienced during plume traverses. Further, by reconstructing the sequence of events that led to the failure of the third flight, we identify a number of lessons learned and propose appropriate recommendations to reduce risk in future flight operations.

## 1. Introduction

The application of instrumented small UAS (Unoccupied Aerial Systems), or alternatively “drones,” has had a transformational influence on volcanological research over the past decade, particularly in recent years where the miniaturization of scientific instrumentation has begun to approach the rapid progression of UAS technology (Jordan, 2019; James et al., 2020). Driven largely by the consumer market, UAS control systems and hardware have now advanced to the point where relatively little training is required to operate multi-rotor platforms equipped with complex sensors. Aerial robotic systems are being developed and deployed increasingly for a range of environmental applications (Fladeland et al., 2011; Vivoni et al., 2014; Detweiler et al., 2015; Klemas, 2015; Pajares, 2015; Bhardwaj et al., 2016). In particular, significant traction is being realized in the areas of remote sensing (Immerzeel et al., 2014; Tamminga et al., 2015), mapping 2D/3D structures (Nagai et al., 2009; Stöcker et al., 2015; Zweig et al., 2015) and atmospheric sampling (Cassano, 2013; Villa et al., 2016; Greatwood et al., 2017) using a range of emerging sensor technologies (Wildmann et al., 2013; Detert and Weitbrecht, 2015; Hill and Clemens, 2015). Atmospheric sampling has been performed either by multi-rotor UAS at lower altitudes in the 500–1,000 m range (Cassano, 2013; Peng et al., 2015) or by fixed wing platforms capable of long-range flight but that require considerable resources to deploy (Ramana et al., 2007; Corrigan et al., 2008; de Boer et al., 2016). The greatest limitation to multi-rotor UAS is often the battery technology, which determines the flight time and therefore distance and altitude (flight envelope). The use of fixed wing UAS can increase the flight time for a given payload and Maximum Take-Off Weight (MTOW), but with additional challenges in terms of launch and recovery, particularly in remote locations and vegetated/mountainous terrain typical of volcanic environments.

In volcanology, remote measurements using UAS now enable the collection of scientific data in previously inaccessible volcanic plumes (McGonigle et al., 2008; Shinohara, 2013; Di Stefano et al., 2018; Liu et al., 2019), or where large areal coverage is required (Darmawan et al., 2018; Favalli et al., 2018), whilst prioritizing the safety of the operator. To this end, aerial observations are now becoming integrated routinely within volcanic crisis response procedures (Turner et al., 2017; Nadeau et al., 2018; de Moor et al., 2019; Syahbana et al., 2019). Notably, most volcanological operations are typically conducted within Visual Line Of Sight (VLOS) and at relatively low altitudes. Critically, however, there remain significant gaps in our knowledge of some of the most active, yet inaccessible, volcanoes where Beyond Visual Line Of Sight (BVLOS) operations are the only way to obtain the data required (Schellenberg et al., 2019; Syahbana et al., 2019; Liu et al., 2020). Here, we focus on BVLOS operations at Manam volcano, Papua New Guinea, in the context of an international scientific effort to characterize the chemistry of the volcanic gases being released from this globally significant volcanic emission source.

Specifically, we present a detailed account and analysis of the platform development (“Titan” SUAS) and the operational procedures needed to realize safe and repeatable operations to an altitude of 2,300 m Above Mean Sea Level (AMSL) and a horizontal distance of 5 km from the take-off point. We analyse logged flight data for three flights to explore parameters related to aircraft performance, turbulence within the volcanic plume, and energy budgets. Although scientific data were collected from all three flights, we critically evaluate the event sequence that resulted in loss of the airframe during the third flight. Through the lessons learned and insights into plume conditions presented, our results will contribute to the continued development and operation of robust fixed wing sensor platforms for the volcanological community, and in extreme environments more generally.

### 1.1. Motivation

Measurements of volcanic gases are critical for the assessment of volcanic hazard (Aiuppa et al., 2007; de Moor et al., 2016, 2019) and for constraining global emissions of environmentally-important gases, such as carbon dioxide (Aiuppa et al., 2019; Fischer et al., 2019; Werner et al., 2019). Volcanic environments present challenging environments in which to make scientific measurements, particularly at high altitude, densely vegetated, or highly active volcanoes. These sampling limitations have led to significant bias in estimates of global volcanic gas emissions toward a relatively small number of accessible, passively degassing volcanoes (Fischer and Aiuppa, 2020). By enabling proximal sampling of remote or hazardously accessible volcanic plumes, instrumented UAS are now targeting gaps in our knowledge of gas emissions at some of the major remaining “known unknown” volcanic emitters.

**Figure 1:**
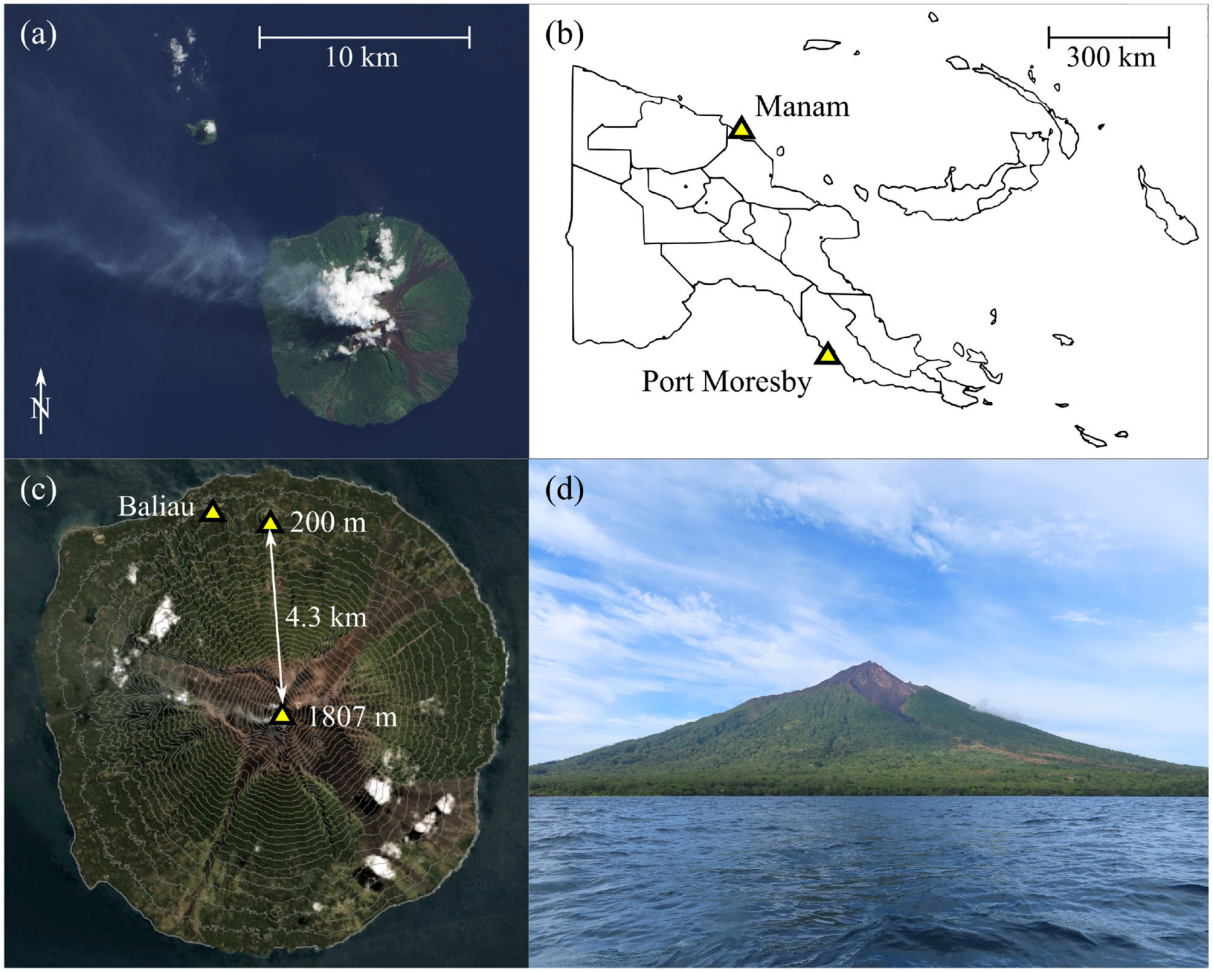
**(a)** Satellite view of Manam with a visible plume drifting North-West. **(b)** Manam volcano is located on the Northern coast of mainland Papua new Guinea. **(c)** Each mission required flying to the summit from a small satellite cone located 4.3 km from the summit crater, near the village of Baliau. Note the satellite image has been overlayed with contour lines indicating the steep terrain. **(d)** A clear view of Manam volcano from the approach by boat.

Manam (Figure 1) is one of the most active volcanoes in Papua New Guinea (Palfreyman and Cooke, 1976), and has experienced five major eruptions in the past year alone (GVP, 2019). A series of climactic eruptions in 2004 devastated large sectors of the island and displaced the local population to the mainland. Mild to moderate explosive activity has continued sporadically at Manam since the 2004–2006 eruptions, causing continued social and environmental disruption (Mercer and Kelman, 2010). In a broader context, Manam is a globally-significant source of sulfur dioxide to the atmosphere (Carn et al., 2017) as measured by satellites, and yet its carbon dioxide emissions are previously uncharacterized. Aerial-based Observations of Volcanic Emissions (ABOVE), of which this study is a part, is an internationally-collaborative and cross-disciplinary endeavor to integrate novel UAS technology with state-of-the-art gas sensing instrumentation to improve our ability to measure the gas chemistry and emission rate at remote and inaccessible volcanoes, such as Manam. In this contribution, we focus on the engineering and control required to achieve long-range, high altitude fixed wing flights through the volcanic plume. The resulting scientific data are presented in a companion publication (Liu et al., 2020).

The requirements of volcanic plume intersection, at long-range or otherwise, present considerable challenges in terms of both hardware engineering and flight control. Volcanic plumes are energetic and thermally-buoyant mixtures of gas and (sometimes) ash particles, which are often emitted in a pulsatory manner (Woods, 2010). In the case of Manam, the plume can rise to altitudes of several kilometers above the vent under its own thermal energy before dispersing laterally with the wind (Liu et al., 2020). Yet, our knowledge of the conditions encountered within a volcanic plume is incomplete, leading to considerable uncertainty when designing an appropriate air frame and optimal flight path. To achieve plume intersection, we developed an instrumented fixed wing (7.5 kg UAS, hereafter referred to as the “Titan” aircraft). The Titan system is capable of carrying a payload of 1 kg up to an altitude of 2300 m ASL, for a distance of more than 10 km. The aircraft is also capable of a hand launch in zero wind, which combined with a parachute recovery makes it ideal for operation in rough terrain, or areas with very little flat ground for a conventional landing. Quad-plane type airframes can also operate under these conditions, but typically lack the required performance for long range missions with large ascent requirements.

By analyzing flight data from two successful flights at Manam, we present novel insight into the atmospheric conditions and the resulting airframe stresses encountered within the extreme environment of the plume of an active volcano. Further, by reconstructing the sequence of events that led to the failure of the third flight, we identify a number of lessons learned and propose appropriate recommendations for future flight operations and aircraft design requirements.

### 1.2. Manam Volcano (Papua New Guinea)

Manam volcano is located 13 km off the northern coast of mainland Papua New Guinea (Figure 1b). Most of the volcano is submerged but the exposed sub-aerial part of the volcanic edifice forms an island ~10 km in diameter. Current volcanic activity involves persistent passive gas release, punctuated by occasional large explosive eruptions (GVP, 2019). A distinctive gas plume is often visible from both the ground and space (Figure 1a). With an almost equatorial latitude, the climate is tropical with temperatures of ~30 °C and frequent rainfall. The flanks of the volcano are often obscured by cloud, especially from late morning through to mid-afternoon, although the summit can be clear above the cloud level.

The topography of the island is generally mountainous with small patches of level ground near the coast. The flanks of the volcano are densely vegetated and incised by four radial avalanche valleys (Figure 1c) that channel debris flows during large eruptions. The summit altitude is 1,807 m AMSL (GVP, 2019). A small volcanic cone (known locally by the name “Godagi”) is located on the northern coast of the island, and has a summit altitude of 200 m AMSL. We selected Godagi cone as the base for fixed-wing operations due to its prominent topographic position, clear lines of sight in all direction unobscured by vegetation, and the altitude advantage. We identified an area of tall grass ~10 × 10 m square as an appropriate landing zone for parachute recovery.

## 2. Instruments and Methods

### 2.1. Titan SUAS

**Figure 2:**
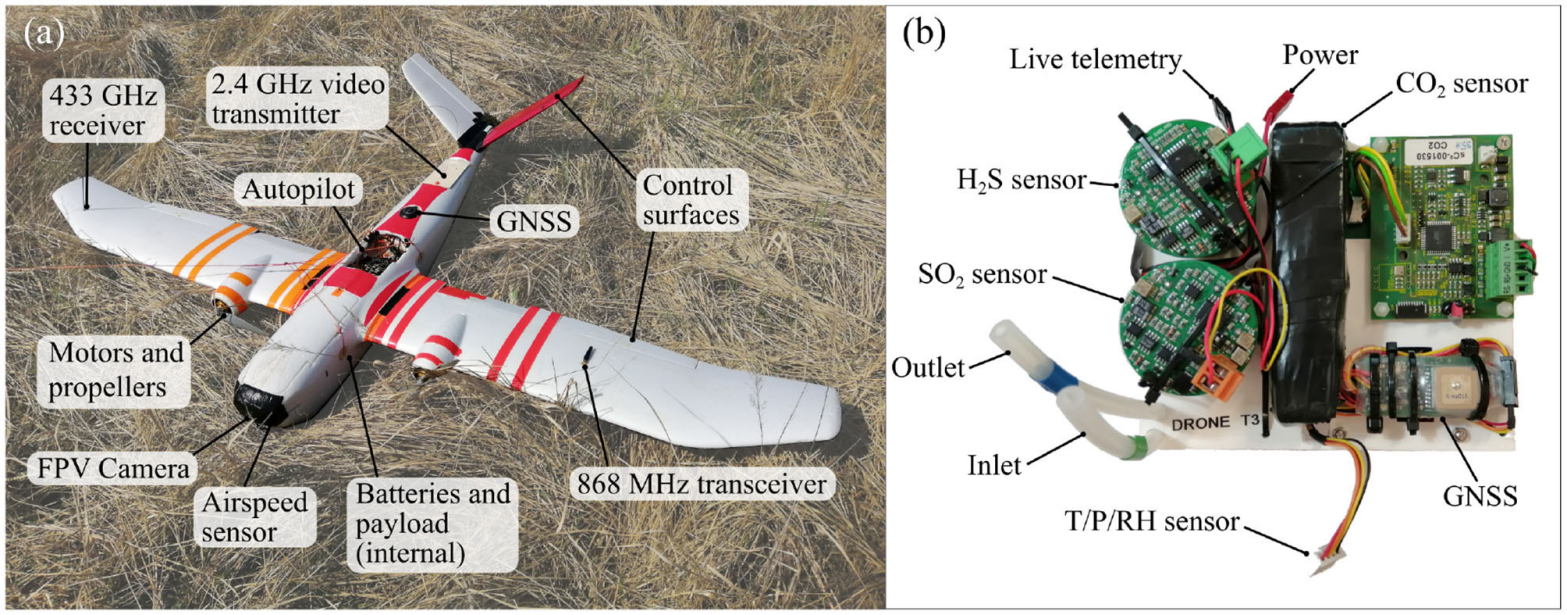
**(a)** The “Titan” fixed-wing UAS. The radio frequency transmitters have been positioned as separated as possible to ensure clear transmissions without blocking from the fuselage or interference. **(b)** The multi-species gas sensor unit. The sensor was additionally shielded with metal foil (not shown) when installed in the fuselage to prevent interference from the vehicle RF transmitters.

The fixed wing platform chosen for this project was the so called “Titan” —a twin-propeller, v-tail vehicle based upon the airframe kit of the same name (Figure 2a). A full list of avionics and specifications is given in Table 1. The aircraft has a wingspan of 2.1 m and a take-off weight of 8.5 kg (including 1 kg payload). This particular system was advantageous because it could be hand-launched and recovered by parachute into confined areas where a “skid” landing would have been impossible. The twin propeller design allowed for the installation of oversized motors which are essential to achieve acceptable climb rates. Power was provided by a 12.75 A h, 6S 22.2 V lithium polymer (LiPo) battery set (comprising three 4.25 A h to allow for international travel), giving a flight duration of 25–35 min depending on each mission's altitude-gain and airspeed requirements—nominally 2,100 m above takeoff, and 18 m s^−1^ equivalent air speed (EAS). The maximum thrust was measured in the laboratory to be 7 kg, hence the vehicle in this configuration had a thrust to weight of 82%. This was essential for the hand launch and to ensure the motors were operating at a sustainable power of ~40% during the long climbs.

**Table 1:** Parts list for the “Titan” aircraft.

**Spec./Part**	**Details**
Maximum flight time	1 h (100 m ascent), 30 min (2,000 m ascent)
Take-off mass	8.5 kg
Wingspan	2,160 mm
Airframe	Skywalker Titan (China)
Battery (×3)	Overlander Supersport Pro 22.2V 4250 mAh 35 C (UK)
Main Motors (×2)	AXi 2826/13 v2 (Czech Republic)
Speed Controllers (×2) (ESC)	Jeti Spin Pro 66 OPTO (Czech Republic)
AutoPilot	UnmannedTech Pixhawk v1 (UK)
Autopilot Software	ArduPlane V3.9.7
Propellers (×2)	APC-E 12 × 6 Thin Electric (USA)
All Servos (×5)	Hitec HS-5065MG Digital (Japan)
Safety (Pilot) control link	DragonLink V3 Advanced (433 MHz) (USA)
Ground telemetry link	RFD 868x (868 MHz) (Australia)
FPV link	ImmersionRC 700 mW (2.4 GHz) (Hong Kong)
FPV Camera	RunCam Eagle 2 Pro (Hong Kong)
Parachute	Skywalker Landing Umbrella 8 kg (China)
Companion computer	PJRC Teensy 3.6 micro-controller (USA)

**Figure 3:**
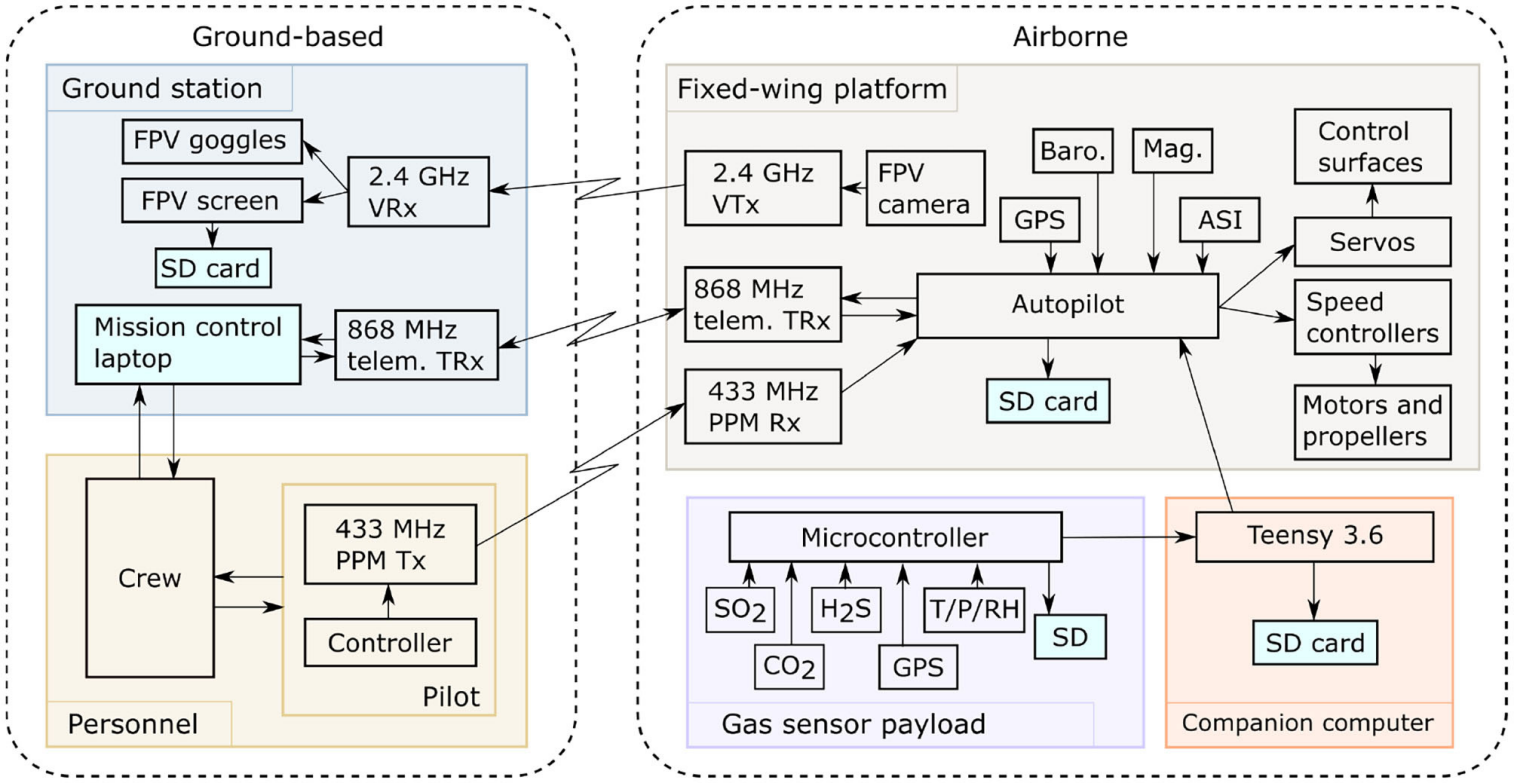
Interconnection of the UAS sub-systems. Live sensor data is relayed from the sensors, via the companion computer, autopilot, and ground station before being displayed live to allow guidance during FBW flight segments.

The Titan featured a full autopilot flight computer with supporting sensors (GNSS, barometric altitude, airspeed indicator, and IMU). Running the open source ArduPlane software, the autopilot was capable of navigating the aircraft along pre-planned waypoint missions. Three wireless links were used to interact with the vehicle during flight. The pilot safety link, operating on the 433 MHz frequency, was used for initializing the automatic flight and for manual control during the plume intersections and parachute landing. The second link was a bi-directional telemetry modem operating on the 868 MHz frequency and was used for monitoring flight statistics, to issue updated commands to the autopilot, and also relay live gas concentration measurements to the ground station. The third link was a live first-person-video (FPV) stream from a camera in the nose of the aircraft operating on the 2.4 GHz frequency. The interconnection of the avionics systems is shown in Figure 3.

There are three internal cabins within the body of the “Titan.” The first is the fuselage, which housed the control systems and batteries. The second is the payload bay, which contained the gas sensor and a downwards-orientated camera. The final cabin, located toward the tail of the aircraft, contained the parachute landing system and video transmitter.

The autopilot logged flight data at frequencies between 10 and 50 Hz, including signals, such as altitude, airspeed, orientation, servo commands, and GPS location. A reduced rate version of these signals is telemetered to the ground control station where they are also stored. Both the high-fidelity onboard and low-fidelity ground station logs can be analyzed post-flight alongside the recorded videos. These log files are often overlooked at the end of a successful mission, however they provide a rich source of additional information when analyzed more thoroughly. In section 4, we extract selected signals for detailed analysis to inform future UAS design requirements.

The ground station comprised receivers for the telemetry and video links, a laptop, a display screen, and pilot controller. The telemetry data are received, decoded, and displayed, with the live gas concentration data shown in a custom application. The live video is displayed and recorded on a handheld monitor screen. All items are battery-powered and portable. During flight, live data streams of parameters, such as battery voltage, airspeed, GPS-location, and gas concentration were monitored by the operation crew, which included a pilot, co-pilot, and payload specialist. The pilot held the safety link controller, which was used to trigger mode changes, deploy the parachute, and maneuver the aircraft manually whilst in Fly By Wire (FBW) mode. When maneuvering manually at ranges beyond visual line of sight, the pilot used a video headset to view the First Person View (FPV) stream and direct the aircraft. The co-pilot monitored the vehicle telemetry data, verbally relaying essential flight data to the pilot for situational awareness, and, whenever necessary, adjusted mission parameters under instruction from the pilot. The payload specialist monitored the telemetered gas concentration data and FPV video, providing guidance on the quality of the data collected and suggesting modifications to the flight path based on the incoming data. Decisions to deviate from the pre-planned mission were agreed by all crew before they were executed.

The payload comprised two miniature high definition cameras and a multi-component gas analyser system (multi-GAS). Two 4K video cameras (120 g each) were installed in the vehicle: one in the nose with a forward view and one in the payload bay with a nadir view. The multi-GAS is a miniaturized version of the established ground-based volcanic monitoring system developed by the University of Palermo-INGV (Aiuppa et al., 2007); (Figure 2b) and described in (Liu et al., 2019). The multi-GAS unit has dimensions of 150 × 130 × 90 mm and a weight of 550 g. Air is sampled from outside the fuselage and passed through a filter, two electro-chemical sensors (SO_2_, H_2_S), and a NDIR sensor (CO_2_) before being expelled back into the freestream airflow. A separate pressure, temperature, and relative humidity sensor was also mounted on the exterior of the airframe to measure the air conditions. All payload data is stored on micro-SD cards, with the gas measurement data additionally telemetered to the ground station using an onboard companion computer to interpret and forward the essential values.

### 2.2. Flight Planning and Deployment

**Figure 4:**
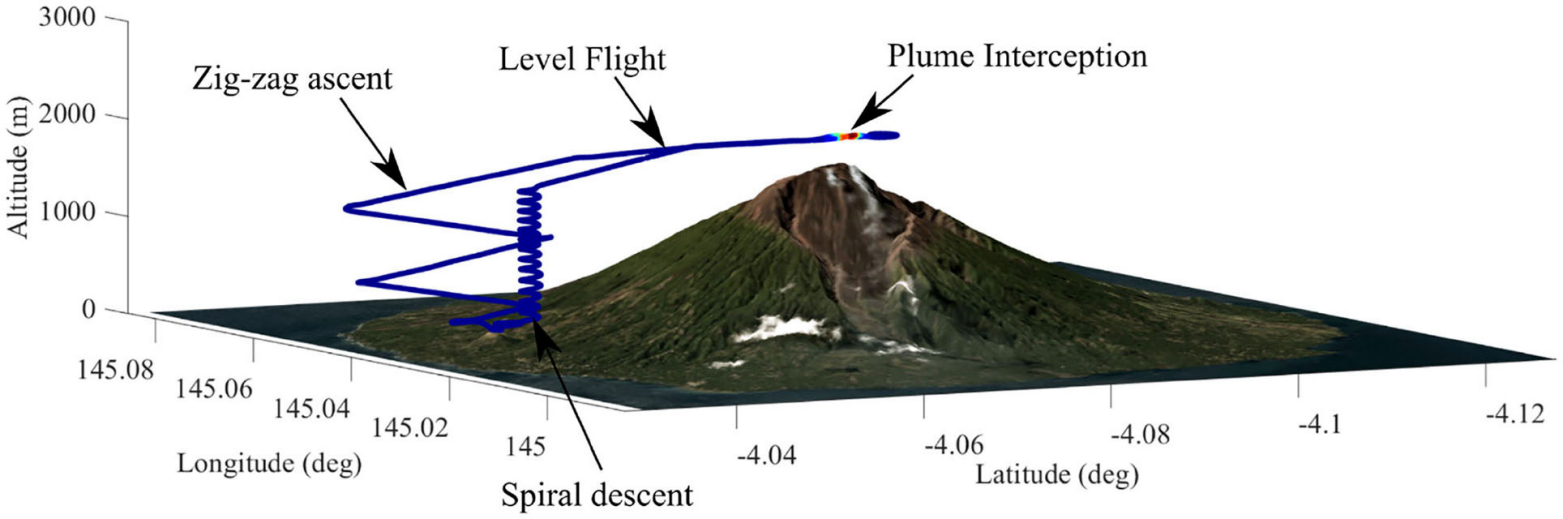
The mission profile comprised a zig-zag ascent, level flight during the plume interceptions, and a spiral descent.

Flight operations included both automatic and manual flight segments. Initially, each mission was pre-programmed as a series of 3D waypoints based upon visual observations and coordinates taken from a high-accuracy digital elevation model (WorldDEM provided by Airbus Defense and Space GmbH). For reference, the coordinates of the take-off location and summit were [−4.0407N, 145.0356E] and [−4.0776N, 145.0384E], respectively, which are separated by ~4.3 km horizontal distance and 1600 m vertical ascent. The Titan has a proven performance history for long-range missions having previously been deployed for low altitude survey missions where a flight duration of 1 h was achieved with a similar payload mass (Connor et al., 2020). Here, the expected flight duration was reduced to ~30 min to accommodate the increased power consumption during the initial climb flight segment. A typical volcanic gas sensing mission is shown in Figure 4.

The flight segments were:

1. **Automatic take-off:** The vehicle is hand-launched, during which the auto-pilot keeps the wings level and the vehicle at full thrust until an ascent threshold of 15 m is achieved. At this point the aircraft has achieved cruising air-speed and begins the waypoint mission.
2. **Main ascent:** The auto-pilot guides the aircraft along a series of large “zig-zag” ramps. The ramp angle is set in the range 10–13° which is an acceptable indefinite motor load (<50% throttle, 40 A) for the hardware chosen. The final waypoint of the ascent is set at the desired plume intercept altitude, and has a horizontal offset of at least 1 km from the summit to ensure the aircraft enters a steady straight and level fight clear of any terrain. The summit overflight altitude is planned very conservatively to minimize risks due to inaccuracies in terrain data or poor weather conditions.
3. **Plume Intercept:** there are two options:
  - **Automatic:** If the pre-programmed mission successfully intercepts the plume, the auto-pilot is left in automatic mode (hereinafter referred to as AUTO) to perform a series of back-and-forth transects. Successful plume interception is identified in real-time by a rise in SO_2_ concentrations, which are monitored at the ground station.
  - **Manual:** If the vehicle fails to intercept the plume, the pilot can choose to take manual control of the aircraft with fly-by-wire mode (hereinafter referred to as FBW), using the live video stream from the forward facing camera to visually direct the vehicle toward the plume. Essential flight statistics (airspeed and altitude) are relayed to the pilot by the ground station operators.
4. **Descent:** After the plume transects are completed or a low-battery threshold is reached, whichever occurs first, automatic flight is resumed for the descent. The descent profile is a large spiral path (Figure 4) to an altitude of 60 m above the landing point. The vehicle then circles the landing point indefinitely until the pilot resumes control. In this environment, the decent can be as steep as required since the power requirements are minimal, however, must still be within the stable flight envelope.
5. **Landing:** The vehicle is recovered using a deployable parachute. Due to inaccuracies in GNSS positioning and drift of the barometric altimeter, this process is flown manually by the pilot who aligns the vehicle over the landing zone (flying upwind), cuts the throttle (to avoid the lines being caught in the propellers), and triggers the parachute release. The descent rate is ~5 m s^−1^ until touchdown and the airframe is sturdy enough to width-stand impact on hard ground.

Full permissions were issued by the Civil Aviation Safety Authority of Papua New Guinea (CASA PNG) with exemptions issued for Beyond Visual Line of Sight (BVLOS) operations at altitudes above the summit height. A Notice to Airmen (NOTAM) was also in place during the entire expedition period to ensure other airspace users were aware UAS were operating.

## 3. Results

Three BVLOS flights over the summit were conducted: one (flight A) on 22 May 2019 and two (flights B and C) on 23 May 2019 (see Table 2). The timings of the flights were in part dictated by when the summit was clear of meteorological cloud—generally either morning, or late afternoon. All flights had pre-planned waypoint missions with a maximum altitude of 2,100 m above the take-off location and a path directly over the summit. This altitude was chosen to place the vehicle ~600 m above the summit, as the buoyant plume typically ascended vertically for several kilometers before dispersing laterally with the wind.

**Table 2:** Details of the three flights.

**Flight #**	**A**	**B**	**C**
Date	22-05-2019	23-05-2019	23-05-2019
Local time in PNG (hh:mm)	16:49	09:17	11:48
Duration (mm:ss)	19:58	28:29	12:38
Battery consumption (mAh)	6,510	8,940	5,190
Notes	Auto plume intercept	Manual plume intercept	Vehicle lost

**Figure 5:**
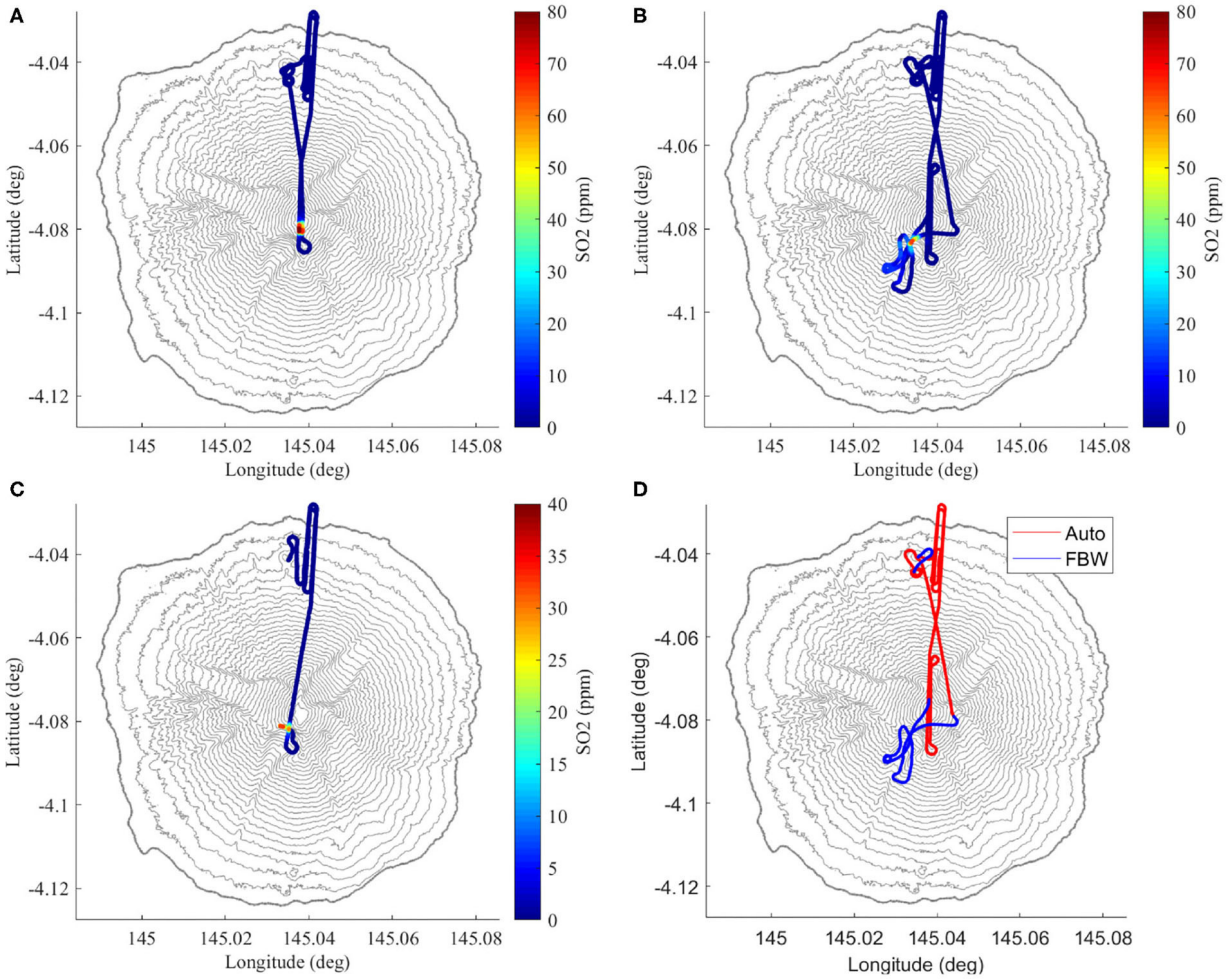
**(A–C)** The three flight paths colored by SO_2_ concentrations. Note how the highest SO_2_ concentrations change location near the summit due to varying wind conditions. **(D)** The changing flight modes for flight B with AUTO (takeoff, ascent) and FBW (plume intercept, landing).

The complete flight paths and measured SO_2_ values are shown in Figures 5A–C. Non-zero SO_2_ values are an indicator of plume interception, due the negligible concentrations in the background atmosphere. During flight A, the vehicle remained in AUTO during the plume transects. The plume was near-vertical at this time, so the flight intercepted the central region of the plume twice. For flight B, the pilot partially used FBW to more accurately penetrate the densest part of the plume for several transects before returning to AUTO for the descent (Figure 5D). The plume was slightly inclined with the wind at this time, meaning the automatic flight path passed tangentially to the plume position. Similarly, FBW was used again during flight C. In this case, however, a failure occurred during the transect and the vehicle was not recovered (section 4.3).

In section 4, we interrogate the autopilot log files to (a) quantify the plume aerodynamic conditions, (b) calculate the energy of the plume up-draft, and (c) decipher the events that led to the loss of the vehicle. For flights A and B, the full-rate log files were downloaded shortly after landing. For flight C, however, only the lower fidelity ground station telemetry log is available.

## 4. Analysis and Discussion

### 4.1. Plume Conditions

Visual observations and theory predict that the conditions within a volcanic plume will be more turbulent than free air, however few data relating to quantification of these conditions exist. Here, we analyse the on-board autopilot sensors to interrogate the plume conditions encountered during flight A in detail.

**Figure 6:**
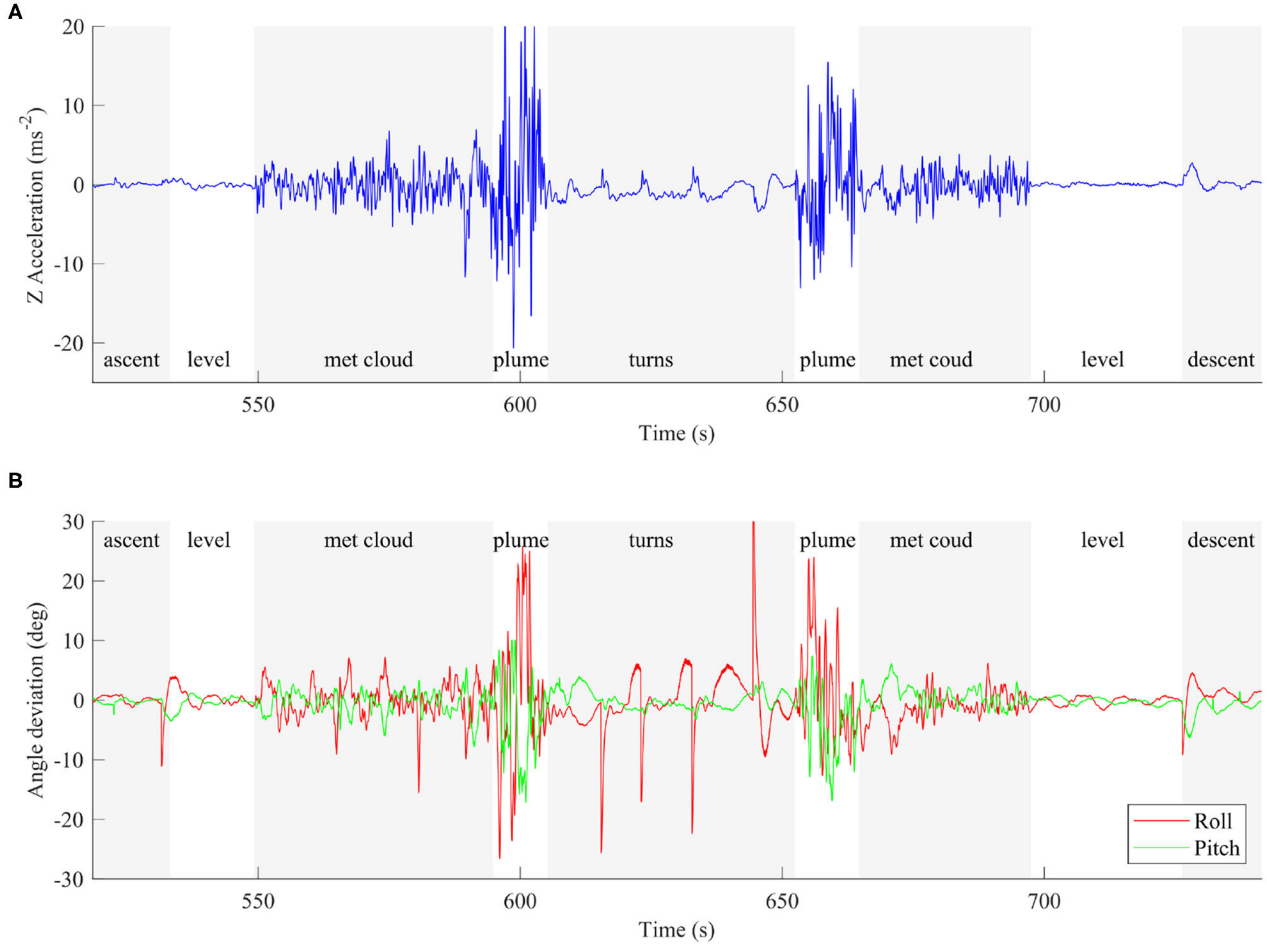
**(A)** The body Z axis accelerations during Flight A. Note the difference in turbulence experienced by the aircraft as it encounters the various air conditions. **(B)** The roll and pitch deviations calculated as the difference between the orientation and its target. Larger values indicate the vehicle has been perturbed further from its trimmed condition.

The body frame accelerations provide a good indication of the conditions the vehicle was experiencing. During the summit overpass in Flight A the vehicle experienced several different air conditions, each characterized by distinctive Z (vertical) axis accelerations. The various segments have been determined by judgement, however the sharp rise in SO_2_ also gives an indication of the main volcanic plume boundaries. Alternatively, methods to automatically determine the plume interception could be applied (Schellenberg et al., 2019). Figure 6A shows time series data for body accelerations during the summit overpass and has been labeled with the various air conditions encountered. A 5 Hz low-pass filter has been applied to remove high frequency noise. Specifically, we filter the data to ensure the accelerations are representative of the whole body accelerations rather than the small motions of the autopilot module on its flexible vibration isolation mount. The vehicle was in a state of straight and level flight (“level”; Figure 6A) for ~44 s, and during this time encountered a maximum acceleration of 0.82 m s^−2^ with a standard deviation of 0.23 m s^−2^. Prior to and following plume interception, the aircraft passed through meteorological cloud surrounding the summit (“met cloud”; Figure 6A). In total, the vehicle was in the met cloud state for ~78 s, encountered a maximum acceleration of 11.7 m s^−2^ and a greater standard deviation of 1.88 m s^−2^ compared to level flight. The two plume traverses (“plume”; Figure 6A) are delimited by a step change in the magnitude of the body accelerations encountered. The vehicle was inside the volcanic plume for ~22 s, during which the maximum vertical acceleration was ~25.1 m s^−2^. This acceleration translates to effectively a 2.5**g** (where hereinafter **g** refers to g-force) additional loading once the offset of gravity (local gravity assumed to be 9.77 m s^−2^) has been accounted for, and the standard deviation increases to 6.89 m s^−2^. Following the first plume traverse, the vehicle entered a turning phase, which involves a wide 180° turn to reverse the flight path. The turn segment is not analyzed in detail since the aircraft was maneuvering actively, hence larger accelerations than level flight are expected.

We also consider the deviation of the body angles away from straight and level flight as a further indicator of plume conditions. Figure 6B shows the time series of the errors, where greater angle deviations represents larger differences in either pitch or roll from the target orientation. For roll this will be wings level, and for pitch it will be the cruise trim attitude. Again, we observe changes in the characteristic of both signals as the aircraft enters the different air masses described above. The pitch is controlled by the autopilot in order to return to the correct altitude, hence is expected to vary when passing though the plume, but the only cause for the roll deviations is turbulence. The roll deviations are 1–2° in clean air, 5–10° in meteorological cloud, and up to 25° in the plume. If vehicle was already turning at its maximum 35° FBW bank angle when a gust hit, it could force the aircraft into a 60° roll angle, which is outside of the tested flight envelope.

Based on the accelerations and attitude deviations observed, the vehicle is using a significant portion of its flight envelope to remain on course. Any maneuvering during a plume transect could add additional loads that move the air-frame and control algorithm outside of the tested flight envelope where failure might occur more easily. It is recommended that all plume transects are in straight lines with turns outside the plume boundaries to ensure the maximum stability and strength margins are available for the most turbulent plumes.

### 4.2. Energy From Plume

The thermally buoyant plume can be considered a source of additional energy to maintain flight. During flight A and B, the aircraft gained altitude when passing through the ascending air mass above the summit vent. To quantify the energy “gained” during the plume traverses we evaluate the total energy deviation of the aircraft, including the sum of the potential and kinetic energy deviations from the expected cruise conditions, and the energy not used by the propulsion system.

The potential energy deviation, *E*_*p*_, was calculated by from the difference between the current height *h* above the target altitude *h*_*C*_, hence:

(1) $$\begin{array}{l} E_{p}=mg\left(h-h_{C}\right) \end{array}$$

The kinetic energy deviation, *E*_*k*_, is calculated as the difference between the energy at cruise speed (*V*_*C*_) and the current airspeed (*V*_*T*_). Note the altitude-adjusted true air-speed (TAS) is used since this is the speed of the vehicle relative to the air-mass (Jimenez et al., 2017).

(2) $$\begin{array}{l} E_{k}=\frac{1}{2}m\left(V_{T}^{2}-V_{C}^{2}\right) \end{array}$$

We also account for the energy consumed by the motors, since any increase in altitude or speed may be due to increased thrust and not plume buoyancy. The power consumed (or not consumed) by the motors is calculated from the measured current (*A*_*p*_) and voltage (*V*_*p*_). By integrating the difference between the current power consumption and a cruise power condition (*P*_*C*_), we can compare this parameter directly to the potential and kinetic energies derived above. The cruise power condition is found by averaging the power consumption during straight and level flight segments outside of the plume. The integral is taken over the time period (*t*_1_) to (*t*_2_) (annotated on Figure 8), corresponding to a subsection of the total flight from when the aircraft has finished the ascent to immediately before the descent commences.

(3) $$\begin{array}{l} E_{m}=\int_{t_{1}}^{t_{2}}\left[\left(V_{p}A_{p}\right)-P_{C}\right]dt \end{array}$$

**Figure 7:**
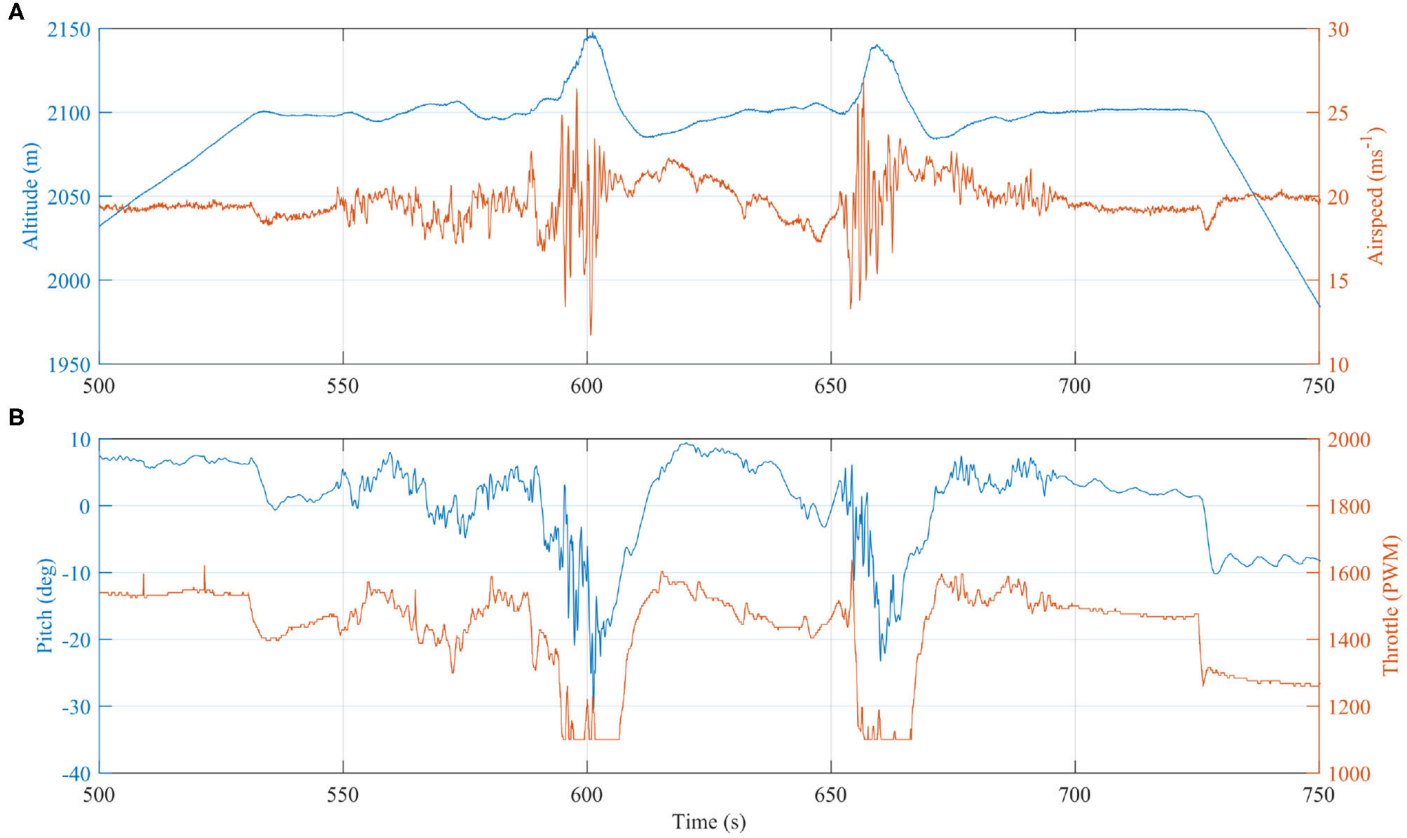
Time series of **(A)** altitude and airspeed, and **(B)** throttle and pitch, with a common time axis as the vehicle encountered the rising plume. Due to the hot rising buoyant plume air mass, the vehicle gains altitude despite reducing throttle to zero and orientating nose down with a negative pitch angle.

**Figure 8:**
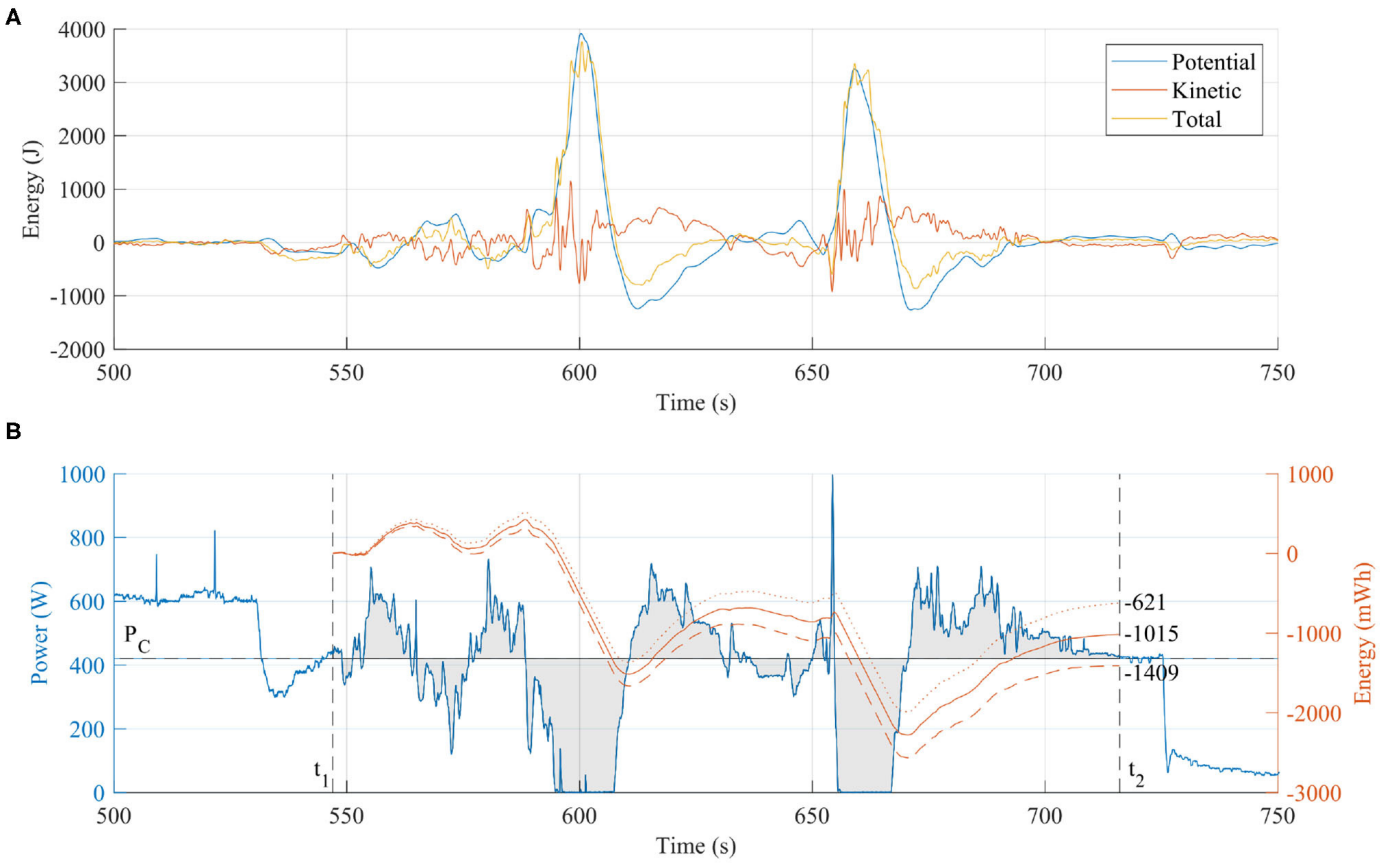
**(A)** The kinetic and potential energy are almost equal before and after the plume transect because the autopilot corrected errors in speed and altitude. However, less electrical energy was consumed due to the plume updraft. **(B)** By integrating the power consumed during the plume transect period, we can calculate the battery energy “saved” in mWh. The effect of small variations (±2%) in the nominal cruise power, *P*_*C*_, are shown by the dashed and dotted lines, respectively in **(B)**, which indicate the method is sensitive to this value.

Auto mode was engaged during plume traverses, during which the flight computer attempts to maintain course, speed, and altitude. Figure 7 shows time series data for altitude, throttle, airspeed, and pitch on a common time axis for the first transect. Airspeed is maintained at ~20 m s^−1^ TAS and the throttle is cut to zero (indicated by a PWM value of 1,100) whilst the autopilot demands a nose down pitch at the maximum FBW angle of −25°. Yet, despite the autopilot response, the vehicle still ascends by ~45 m above the target altitude, therefore gaining energy in a similar manner to a glider loitering in a thermal. Once the vehicle emerges from the plume and returns to less turbulent air, the autopilot energy control algorithm successfully returns the aircraft to cruise at the set speed and altitude. This is apparent from Figure 8A, where the potential and kinetic energy of the aircraft return to the outside of plume value. The reduction in throttle, however, was significant during the transect with the main motors stopped allowing the aircraft to briefly glide. Even with the varying throttle commands, when the total power consumption deviation is integrated over the time interval, there is an overall reduction of energy consumed compared to that consumed if the aircraft had flown in clean air. Figure 8B shows the power consumed and a cumulative integral of the difference between the current power and assumed cruise power of *P*_*C*_ = 420W. The final value of the integration is *E*_*m*_ = −1, 015 mW h where the negative sign indicates an energy saving. This method is sensitive to the selected value of *P*_*C*_, therefore the integral was also calculated for *P*_*C*_ = 420 ± 2% resulting in *E*_*m*_ = −1, 015 ± 394 mW h. This equates to 44 mA h battery capacity with an assumed 22.5 V battery voltage.

Although the energy saved is only a small proportion of the total energy consumed, this evaluation demonstrates quantitatively the potential to harvest energy from a volcanic plume. Optimization of the autopilot response and mission plan could increase further the energy saved, and therefore the harvesting potential. For example, the steep pitch down attitude used to return the vehicle to the set point altitude will have placed the aircraft in an unfavorable aerodynamic state with increased drag losses wasting the potential gains. Also, the turning segment of the flight was planned conservatively, meaning the aircraft could have turned earlier and thereby reduced the time spend outside the buoyant plume. The aircraft is within the plume for 10 s on the first pass and 12 s on the second (Figure 6), which, with a true airspeed of 20.8 m s^−1^, equates to a plume width of between 208 and 242 m. The Titan aircraft has a tightest turning circle of 120 m, therefore it may be possible to loiter within the bounds of the buoyant plume indefinitely. Extending the time spent within the plume is critical to the scientific application (i.e., volcanic gas measurements), where the associated uncertainties are for the most part related to the measurement duration. Differences in sensor response times between gas species introduce uncertainty for derived gas ratios for ground-based measurements (e.g., Roberts et al., 2017), and this effect is amplified for UAS-mounted instruments due to the comparatively short measurement periods (Liu et al., 2019). Response times, in the form of the T90 rise time (the time required for the sensor to equilibrate to 90%, when exposed to a step change in concentration), are generally on the order of tens of seconds for both the electrochemical and NDIR sensors used here. During plume traverses also on the order of tens of seconds, sensors may not have time to approach equilibrium, thus resulting in a signal that is truncated in amplitude relative to the true signal. Harvesting the thermal energy from the plume to extend flight endurance is a critical avenue for future research, and is especially relevant to long-range BVLOS operations.

Flight B has not been analyzed by the same method because the plume transects were not obvious. This was due to the auto mode missing the dense plume on the first pass and the subsequent FBW mode, which produced a more erratic flight path. The increased time spent intercepting the plume causes the power analysis integral analysis to becomes even more sensitive to the assumed value of *P*_*C*_. This increases the errors to an unacceptable magnitude to be confident in drawing conclusions from the data.

### 4.3. Loss of Aircraft

An aircraft was lost during flight C, unfortunately. By analyzing the ground station telemetry logs, we infer the potential cause of failure, in particular, the conditions encountered in the volcanic plume and the order of events. The conclusions presented are somewhat speculative due to the limited data available, however the identified lessons learnt are still valuable for planning future operations and setting requirements for future airframe designs.

We initiated flight C as soon as possible following the successful landing of the previous flight to ensure comparable cloud conditions over the summit. Turn around time was 1 h, with tasks including downloading the sensor data files, swapping battery packs, repacking the parachute, and completing all pre-flight checklists. Visually, plume conditions did not appear to change between the two flights.

**Figure 9:**
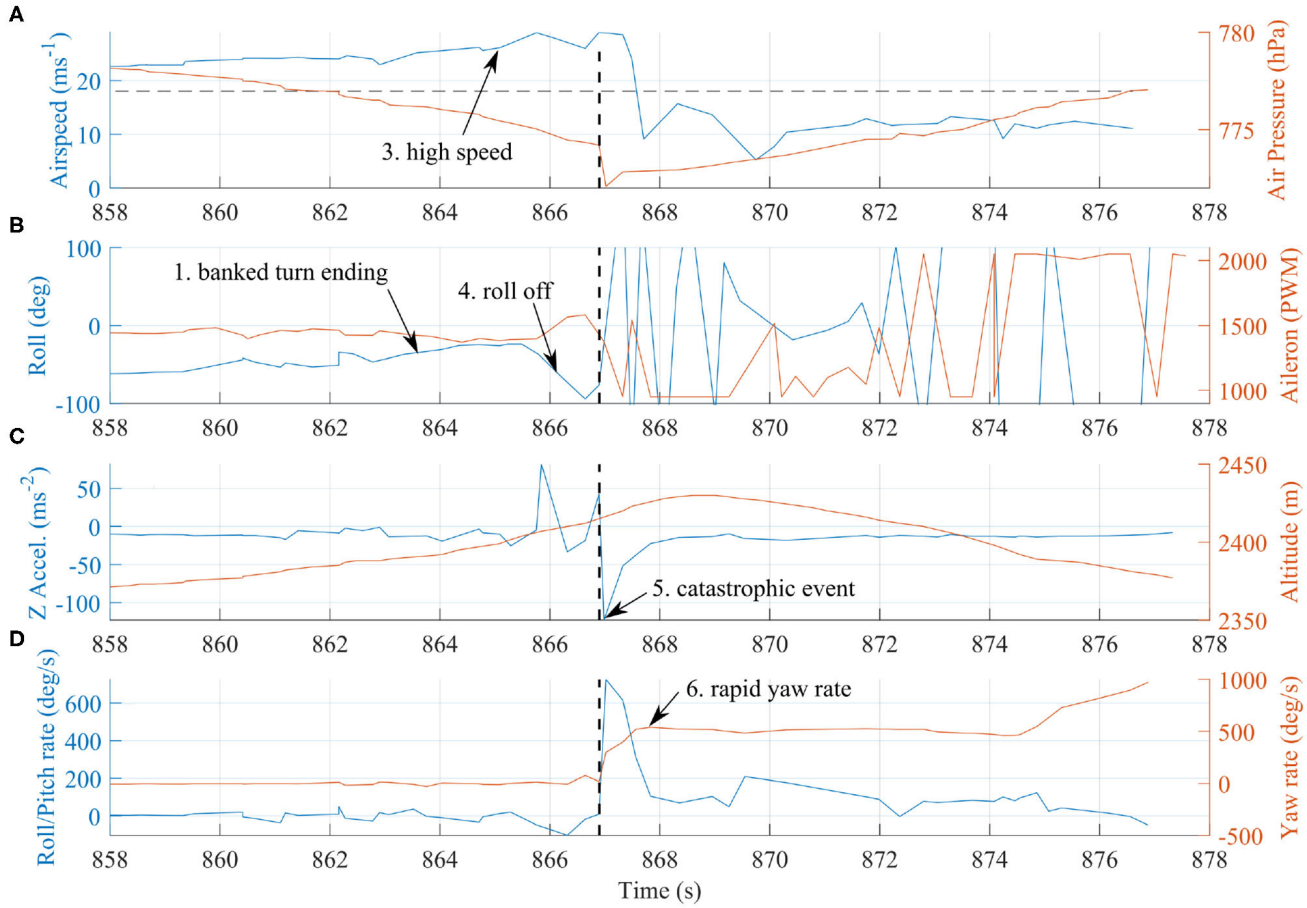
Indicative signals from the telemetry log file of flight C for the final 20 s of transmission. Signals **(A)** airspeed and air pressure, **(B)** roll angle and aileron input command, **(C)** vertical acceleration and altitude, and **(D)** roll, pitch, and yaw angular rates, have been plotted against a common time-axis. A catastrophic event occurs at 867 s followed by a rapid (>1 Hz) spin. See Table 3 for further details of the order of events.

The flight began with an ascent profile and plume approach identical to the previous flights (Figures 5A,B). After passing tangentially to the plume during the first traverse, as in flight B, FBW mode was engaged to guide the direction of the aircraft into the densest region of the plume. However, on re-entering the plume after the first manual turn, there was a catastrophic event that triggered the sequence that ultimately led to the loss of the vehicle. Referring to Figure 9 and Table 3, we highlight key indicators that reveal the sequence of events.

**Table 3:** Summary of the sequence of events that led to loss of the vehicle during flight C. Time is reported relative to takeoff time and is equivalent to Figure 9.

	**Time (s)**	**Event**
1.	858.0–865.0	Vehicle finished a relatively high speed (23 ms^−1^) turn and was returning toward wings level.
2.	865.0	Due to the higher airspeed, the aircraft climbed ~40 m above the cruise set-point, but was near level in pitch orientation.
3.	865.0–867.0	In the 2 s period immediately before the event, the vehicle increased in speed to 26 ms^−1^ due to a high throttle command.
4.	865.8	At this point the vehicle experienced a sudden ^~^7G upwards acceleration (71ms^−2^) and started to roll rapidly at a rate of 100° s^−1^. The hypothesis is that at this point the aircraft experienced a significant up-draft from the main volcanic vent. The aileron moves to oppose the motion, but does not have a significant effect.
5.	867.0	There are several indicators of a catastrophic event. The body vertical acceleration suddenly changed sign and magnitude to ^~^11G (-113ms^−2^). There is also a sudden change in static air pressure, and an abrupt reversal in roll rate.
6.	867–868	Very rapid yaw rate develops indicating a flat spin. Observations from the live video link also confirmed the vehicle was no longer maintaining orientation.
7.	867–877	The main telemetry link was lost 10 s after the event. The vehicle was still high above the summit at this time and falling at ^~^11 ms^−1^. The aircraft's altitude was still high enough for a clear line of sight hence the loss of telemetry is thought to be due to the aircraft breaking up. In this case, we suggest the loss of a wing containing the telemetry module.

Although the vehicle was not in a trimmed level flight condition, it was also not in a dangerous maneuver at the time of the event. We therefore suggest that the loss was caused by a sudden, high magnitude, change in external conditions. Real-time SO_2_ concentrations exceeded 30 ppm immediately prior to the failure (Figure 5C), and therefore we confidently conclude that the aircraft had entered the main region of the plume column. In light of this, the most likely cause of the large vertical acceleration is an energetic up-draft of thermally-buoyant gas from the main volcanic vent. Nadir images from a summit overpass during flight A confirm the presence of shallow magma within the vent crater (Liu et al., 2020). Further, visual observations throughout the field deployment indicate that gas was emitted from the vent in a pulsatory manner, with sporadic pulses of fast-ascending gas superimposed on a background stable emission. At volcanoes where the magma viscosity is sufficiently low to allow decoupling between rising gas bubbles and the magma (as is the case at Manam), outgassing takes place either passively, where gas simply exsolves from the surface of the magma body, or actively, where large bubbles of overpressured gas burst more explosively at the surface (e.g., Edmonds, 2008; Tamburello et al., 2012). Pulsatory gas emissions are common to many volcanoes; the time scale of the periodicity reflects the fluid dynamics that govern gas bubble ascent (Pering et al., 2019). At Manam, the pulses of energetic, thermally-buoyant gas associated with active outgassing would have generated transient conditions of extreme turbulence within the plume column. We propose that entering one of these up-drafts over-stressed the airframe and ultimately resulted in the loss of either a wing-tip or one of the V-tail stabilizers. The loss of either component would generate significantly unbalanced aerodynamic forces, consistent with the rapid rotations indicated by roll and yaw rates. The video link continued transmission for a significant time after the loss of telemetry, suggesting multiple stages of failure following the initial event.

From this investigation we present recommendations for the required aircraft strength based upon the loading encountered. The ^~^7**g** up-gust was the most likely cause of failure, therefore applying a reserve factor of 2 produces a requirement of 14**g** upwards load. A reserve factor of 2 is greater than the typical 1.5 used within aerospace design processes, however there are very limited data available quantifying volcanic plume conditions, hence a greater reserve margin is preferred. The downward load can be more conservative since the aircraft is not expected to be flown inverted or have significant down-gusts. The speed of the aircraft was within the range of values under which the vehicle had been tested, however the additional wing loading from such high speeds would have reduced the structural strength reserve. Consequently, a second operational recommendation is a more advanced FBW control system that maintains altitude and speed, but allows the pilot to “drive” the aircraft's direction.

Key recommended design criteria have been summarized in Table 4. These criteria are based upon the numerical values derived during the above analysis, the vehicle setup parameters, and from experimental field experience.

**Table 4:** Recommended design requirements for fixed-wing UAS, applied to volcanic plume measurements.

**Requirement**	**Description**
Payload capacity	Minimum 1 kg to accommodate high sensitivity instrumentation and appropriate shielding. With careful design iterations a sensor mass might be minimized below this value, however this is a general starting recommendation for airframes of this size.
Ascent capability	Minimum 500 m above the summit height. This allows for conservative flight planning and also mitigates potential variations in the height requirements. The exact height of a volcanic summit might be unknown, or the activity may have modified the summit topography since the most recent survey. Variations in air density due to weather might also reduce the maximum ascent capability of a vehicle.
Airframe strength	14G. This value was determined from the conditions encountered in Flight C including a reserve factor of 2 applied. Structural strengthening should be applied to areas of load concentration, such as the main wing, and where control surfaces attach to the fuselage.
Airspeed capability	Minimum 20 ms^−1^. A reduced airspeed could be advantageous to allow the aircraft to pass through the plume more slowly and therefore collect more data points. However, strong, topographically-enhanced winds can occur around prominent volcanic peaks, hence the top speed of the vehicle must be fast enough to overcome these. Note that the windspeed measured at the takeoff location can often be lower than that encountered at plume altitude.
Structure	At least partial sealing to minimize airflow over the flight avionics. Volcanic plumes contain acidic gases at high humidity, which cause corrosion and failure of electronics. Sealed enclosures may not be possible for components requiring airflow cooling (e.g., main motors), so these items should be inspected regularly.
Flight control modes	Automatic for large ascents to maintain optimal trim conditions, but with a visually-guided (First Person View; FPV) Fly By Wire option to make course modifications that ensure plume interception, as necessary. Once the aircraft is more than 500 m away from the pilot, it is unlikely they will be able to operate using eyesight alone. Augmenting the pilot situational awareness via FPV and flight telemetry data is important for accurate maneuvers and rapid response to unexpected situations.
Maximum roll angle	The maximum roll angle has a direct impact on the structural loading during turn maneuvers. By reducing the maximum roll angle, the loads created during maneuvers can be reduced, leaving a greater strength margin for unexpected turbulence-based loads. However, a reduction below <20° would compromise the handling qualities for manual flight control, hence a balance must be found. It is also recommended that plume transects only be attempted when in straight and level flight. For the Titan aircraft, the AUTO and FBW flight modes were set with a maximum roll angle of 45°.

## 5. Conclusion

Volcanic environments present many challenges for aerial robotics, from the vehicle design through flight planning to the conditions encountered during the flight itself. Yet, despite these obstacles, instrumented UAS are stimulating transformative advances in volcanological research, motivating further engineering development to respond to these challenges. Here, we describe a series of fixed-wing flights BVLOS over the summit of Manam volcano, Papua New Guinea, to measure real-time gas concentrations within the volcanic plume. Our aim was to collect data that would constrain the emission rate of environmentally-important volcanic gases, such as carbon dioxide. However, the insights contributed by this study are also relevant more generally to other plume sampling applications. Specifically, we show that (a) the “Titan” aircraft is a versatile aircraft suited to BVLOS missions in difficult terrain; (b) an air frame can reasonably expect to be subjected to a 2.5**g** loading when traversing a thermally-buoyant volcanic plume, and that this may increase to 7**g** in more extreme, but transient, cases; and (c) energy harvesting from the volcanic plume presents a tractable means to enhance flight endurance, and thus extend the duration of scientific measurements. We describe the physical parameters and propulsion systems used in our aircraft design at a level of detail sufficient to guide future air craft design, and recommend that vehicles are strength tested up to 14**g** to ensure a factor of 2 reserve against the upper end-member condition. Further, for large plumes, such as that encountered at Manam, the flight path could be optimized to ensure maximum additional energy gain. From on-board flight parameters, we reconstruct the sequence of events that ultimately led to catastrophic vehicle failure and attribute the cause of failure to interaction with an energetic thermal updraft from the main volcanic vent, which is a prevalent characteristic of outgassing at similar volcanoes globally and therefore needs to be taken into account during full systems testing. If recent trends continue, scientific applications will increasing look to aerial robotics to enable sensor placement in hazardous environments. The large spatial scales and shifting targets involved (i.e., the plume is in constant motion, vertically and often horizontally) make FBW mode essential to ensure optimal data collection. Therefore, looking forward, the development of FBW modes with more autonomy for the speed and altitude loops, or the introduction of plume-hunting algorithms capable of processing sensor data in real-time for complete automation will be critical to continued advance in this field. Further, the use of quantitative ground-based measurements of plume parameters (for example, plume rise speed, transport direction, and dimensions) to inform flight planning would contribute to both risk reduction and flight efficiency.

## Data Availability Statement

The raw data supporting the conclusions of this article will be made available by the authors, without reservation.

## Author Contributions

KW, EJL, and TR drafted the manuscript. KW, EJL, TR, RC, and JF built and operated the UAS during the field work. AA, GG, and MB developed the gas sensor, supported its integration into the UAS, and contributed to data analysis. KM and II supported the data collection and assisted with in-country logistics. All authors were involved in the data collection, read and revised the manuscript, and approved the submitted version.

## Conflict of Interest

The authors declare that the research was conducted in the absence of any commercial or financial relationships that could be construed as a potential conflict of interest.
